# Outpatient interface challenges for drug safety in Parkinson’s disease patients: a questionnaire based cross-sectional study

**DOI:** 10.3389/fneur.2025.1563636

**Published:** 2025-06-19

**Authors:** Stephan Greten, Clara Niesmann, Lea Krey, Johannes Heck, Florian Wegner, Martin Klietz

**Affiliations:** ^1^Department of Neurology, Hannover Medical School, Hannover, Germany; ^2^Hannover Medical School, Institute for Clinical Pharmacology, Hannover, Germany

**Keywords:** drug safety, outpatient care, interdisciplinary communication, Parkinson’s disease, geriatric patient

## Abstract

**Background:**

Parkinson’s disease (PD) is a chronic and multifaceted disease with a variety of motor and non-motor symptoms. The safe symptomatic drug therapy of often multimorbid patients places enormous demands on the competence, communication and coordination of the treating physicians, particularly in the outpatient sector.

**Objectives:**

This study aimed to explore aspects of drug safety and interdisciplinary communication in the outpatient sector of PD patients.

**Methods:**

A semistructured questionnaire was designed addressing various aspects of drug safety in the outpatient setting. The questionnaire was sent to a total of 1,002 general practitioners (GP) and 1,005 neurologists (NEU).

**Results:**

One hundred and forty-seven NEU and eighty-four GP answered the questionnaire. Overall, NEU treated more PD patients, while GP cared for more geriatric PD patients, especially outside of the outpatient clinic (home visits, nursing homes). Regarding the execution of recommended laboratory or technical check-ups, as well as the prescription of new medications, neither a formal agreement nor structured communication existed. Merely the identification of potential drug–drug interactions (DDI) was regularly carried out by both professions.

**Conclusion:**

The inadequate interdisciplinary communication hampers therapy safety and consequently the safety of the vulnerable PD patient group. For this reason, standardized and comprehensive communication mechanisms are urgently needed. Solution approaches may include an individual protected digital health record or integrated treatment networks comprising all professionals participating in the management of PD patients.

## Highlights

- In the outpatient care of PD patients, general practitioners play a vital role alongside neurologists, particularly for patients at home, in nursing homes and for geriatric patients.
- The communication structure between general practitioners and neurologists has gaps in the area of drug safety control mechanisms, which pose a potential risk to patient safety.

## 1 Introduction

Among geriatric patients, neurological diseases are common (1). In this context, patients with Parkinson’s disease (PD), the second most common neurodegenerative disorder with an average age of onset of over 60 years, stand out for several reasons (2, 3). First, prior to the onset of motor symptoms and during the course of the disease, PD patients develop characteristic non-motor symptoms/disease-associated disorders such as constipation, depression, dementia and bladder dysfunction (4). On top of that, PD patients usually suffer from a number of other comorbidities, especially cardiovascular, metabolic and neurological diseases (2, 5). In particular, common comorbidities comprise hypertensive diseases, chronic kidney disease, polyneuropathies and diabetes mellitus (5). Symptomatic treatment of the disease-specific motor and non-motor symptoms, as well as associated disorders and further comorbidities, regularly requires the administration of several drugs contributing to the common occurrence of polypharmacy (≥5 regularly taken drugs) in PD patients (5). Complicating matters further is the fact that a majority of the applied drugs act on the central nervous system and exhibit a broad and distinct spectrum of side effects. Combined, inherent PD-specific features and external treatment-associated factors create a delicate vulnerability for adverse drug reactions (ADR) and potential drug–drug interactions (DDI) in this patient group (2, 6).

Hence, the successful and safe treatment of geriatric PD patients places high demands on drug safety (7). For this purpose, certain control strategies should be implemented when new drugs are prescribed or the existing regime is adjusted during the course of the disease (2). Such safety mechanisms include regular laboratory and electrocardiogram (ECG) checks, screening for orthostatic hypotension, DDI or contraindications, and a gradual increase in dosage (“start low and go slow”) (2, 8, 9). Since the management of the multifaceted symptoms of PD patients calls for an interdisciplinary approach and is predominantly carried out on an outpatient basis, the aforementioned controls only work through efficient communication (10, 11). High standards of treatment safety can only be attained through the comprehensive exchange of information in an interdisciplinarily shared responsibility. Best practices for the management of PD patients are not yet established in Germany. Thus, PD patients in Germany are treated in a broad range of facilities, including general neurologists’ practices, specialized university outpatient clinics, and general practitioners’ (GP) practices (12, 13). Since there’s no standardized approach, any of these professions could potentially diagnose PD, initiate and maintain treatment, as well as provide ongoing care. These ambiguities place high demands on interdisciplinary collaboration between the various parties involved, as inadequate communication can led to different treatment approaches and gaps in the drug monitoring. Until digital information exchange in the form of an individual electronic health record (EHC) becomes available in Germany, GP form an important interface between all specialist professions and are therefore primarily responsible for general health issues, including those affecting PD patients. Moreover, GP play a crucial role within this context in the management of PD patients, particularly in underserved regions with limited access to neurological specialists, such as rural areas in Germany, and in specific patient populations, such as patients receiving home care or nursing care (14, 15).

To the best of our knowledge, no studies have been conducted so far examining aspects of drug safety and interdisciplinary communication in the outpatient management of PD patients. Thus, the aim of this study was to narrow this knowledge gap in the area of outpatient primary care providers for PD patients. For this purpose, we surveyed and analyzed the control mechanisms of drug safety and the interdisciplinary communication at the interface between GP and neurologists using a questionnaire-based data collection.

## 2 Methods

### 2.1 Design of study

The study was designed as a cross-sectional, questionnaire-based study. Experts in the field of movement disorders with particular experience in outpatient care for Parkinson’s disease, clinical pharmacologists with special expertise in drug safety, and a psychologist with extensive knowledge of questionnaire construction were involved in creating the questionnaire used in the study. In addition to general demographic aspects of patient care, the mechanisms of recommended follow-up examinations to ensure drug safety and interprofessional communication were investigated. The survey was addressed to outpatient neurologists (NEU) and general practitioners (GP) in several federal states in Germany (Lower Saxony, North Rhine-Westphalia, Bremen, Hamburg, Berlin, Brandenburg and Saxony-Anhalt). Practices in both rural and urban locations were considered. In order to obtain an adequate reliability of the results, the target number was set to include at least 100 NEU and GP per group. Assuming a response rate of 10%, 1,005 NEU and 1,002 GP were contacted. The physicians received a letter with an attached questionnaire. The survey was conducted from November 2023 to January 2024. Finally, the data of 147 NEU and 84 GP were included in the analysis. There was no financial or other compensation for the participants for answering the questionnaire.

### 2.2 Study questionnaire

The questionnaire was specifically developed by the study authors to address the research questions. A total of two versions were created, one for GP and one for NEU.

The survey included single and multiple choice, as well as free text questions, which were divided into five major topic areas. In the first section, demographic data of the participants, such as age, gender, number of patients to be treated per quarter, location of the practice and length of time in practice, were collected. The second section covered patient-specific information like the number of patients with PD, the proportion of geriatric patients (≥70 years, ≥3 chronic diseases, ≥5 medications) and the proportion of advanced PD patients (Hoehn and Yahr stage ≥3, severe non-motor symptoms, use of advanced therapies, e.g., deep brain stimulation, levodopa-carbidopa intestinal gel infusion) (16). The third and the fourth section contained specific questions on drug safety aspects for patients with PD. The survey was concentrated on typical control mechanisms for increasing drug safety and on communication between the individual professions. With regard to the exploration of communication patterns, the questionnaires differed in two questions: (1) While the GPs were asked whether they took into account the drug suggestions of other specialists in the medication plan, the NEU were questioned who they considered responsible for organizing this very plan. (2) NEU were asked whether the drugs in a summarized medication plan were constantly updated, while GPs were questioned whether dosages of drugs from all specialties in the summarized medication plan are regularly adjusted and monitored. The last section asked for ideas and wishes from physicians concerning the electronic health record (EHC). The responses were anonymous. Overall, the estimated time for questionnaire completion was 10–15 min.

### 2.3 Statistics

For statistical analysis IBM SPSS (Armonk, NY, United States) and for the graphical visualization Microsoft Excel 2016 (Redmond, WA, United States) were used. For metric data Shapiro–Wilk-test was performed to test for normal distribution. Since none of the evaluated variables were normally distributed, Mann–Whitney-U-test was used for the analyses. Categorical data were analyzed by performing Chi-squared test.

## 3 Results

### 3.1 Demographic profile of the participating physicians

The demographic characteristics of the participants are displayed in Table 1. In terms of age (NEU, 53.4 ± 9.1 years; GP, 54.1 ± 9.4 years) and gender distribution (NEU, 45.3% females; GP 46.1% females), as well as the number of active years in medicine (NEU, 25.6 ± 8.7 years; GP, 25.6 ± 9.5 years), NEU and GP were comparable.

**Table 1:** Demographic characteristics of participants.

	Neurologists (*n* = 147)	General practitioners (*n* = 84)
Sex, female, *n* (%)	62 (45.3)	35 (46.1)
Age, years (mean ± SD)	53.4 ± 9.1	54.1 ± 9.4
Time of medical practice, years (mean ± SD)	25.6 ± 8.7	25.6 ± 9.5
Time in outpatient clinic, years (mean ± SD)	12.9 ± 9.0	**17.1 ± 9.6** ^##^
Patients per quarter, *n* (%)		
≤500	13 (9.6)	2 (2.7)***
501–1,000	65 (47.8)	20 (26.7)***
1,001–2,000	49 (36.0)	40 (53.3)***
>2,000	9 (6.6)	13 (17.3)***
Localization of the practice, *n* (%)		
Urban	93 (67.9)	34 (44.7)***
Rural	44 (32.1)	42 (55.3)***

^##^*p* < 0.01, Mann–Whitney-U-test; ***p < 0.001, chi-squared test.

SD, standard deviation. Significant differences are highlighted in bold.

GP were established in an outpatient clinic for significantly longer than the NEU (NEU, 12.9 ± 9.0 years; GPs, 17.1 ± 9.6 years; Mann–Whitney-U-test, *p* < 0.01). Furthermore, the GP saw significantly more patients than the NEU. The majority of GP treated between 1,001 and 2,000 patients per quarter (53.3%), whereas the majority of NEU treated between 501 and 1,000 patients per quarter (47.8%; chi-squared-test, *p* < 0.001). Lastly, the GP’s outpatient clinics were more often located in rural areas than the NEU’s offices (NEU, 32.1%; GP, 55.3%; chi-squared-test, *p* < 0.001).

### 3.2 Characteristics of treated PD patients

NEU, as expected, saw more PD patients in their outpatient clinics than the GPs. 49.6% of the NEU treated more than 50 PD patients, in comparison to only 1.3% of the GPs (chi-squared-test, *p* < 0.001). A significantly higher proportion of NEU than GP stated that at least 25% of their PD patients were in an advanced (NEU, 74.4%; GP, 50.7%; chi-squared-test, *p* < 0.01). The proportion of PD patients fulfilling the criteria of a geriatric patient (≥70 years, ≥3 chronic diseases, ≥5 medications) was significantly higher among GP. Compared to 48.0% of GP, only 27.9% of NEU reported that the proportion of geriatric patients was over 75% (chi-squared-test, *p* < 0.05). NEU and GP differed significantly in the setting of patient care: although both treated the majority of their patients in their outpatient clinics (NEU, 96.6%; GP, 78.6%), GP provided more care in home visits (NEU, 4.8%; GP, 26.2%; chi-squared-test, *p* < 0.001) and in nursing homes (NEU, 26.5%; GP, 31.0%; chi-squared-test, *p* < 0.001).

### 3.3 Drug safety aspects

About two-thirds of the NEU (61.1%) and GP (63.9%) surveyed reported that they were aware of all the drugs prescribed to PD patients. Over-the-counter drugs (OTC) were only recorded by 16.6% of NEU and 14.3% of GP.

Table 2 illustrates the findings regarding potential control mechanisms of drug safety. In our survey, 84.4% of NEU tended to delegate regular ECG checks to GP, while only 4.1% performed these themselves. In contrast, a significantly higher proportion (45.2%; chi-squared-test, *p* < 0.001) of GPs carried out ECG controls independently. The two professions mostly undertook these independently (NEU, 66.7%; GP, 56.0%).

**Table 2:** Drug safety aspects.

	Neurologists (*n* = 147)	General practitioners (*n* = 84)
ECG controls, *n*		
Independent execution	6	38***
Delegation	124	55***
Not responsible	15	1***
Laboratory control, *n*		
Independent execution	98	47
Delegation	61	43
Not responsible	6	3
Frequency of interaction control, *n* (%)		
Every appointment	29 (20.4)	7 (8.6)***
Once a quarter	26 (18.3)	6 (7.4)***
New drug initiation	86 (60.6)	61 (75.3)***
Never	1 (0.7)	7 (8.6)***
Screening for ACS, *n* (%)		
Yes	99 (70.7)	36 (44.4)***
No	41 (29.3)	45 (55.6)***

The questions marked in italics were multiple-choice questions. ***p < 0.001, chi-squared test.

ECG, electrocardiogram; ACS, anticholinergic side effects. Significant differences are highlighted in bold.

99.3% of NEU and 91.4% of GP reported that they regularly checked for DDI. Both professions would do this primarily when prescribing a new drug (NEU, 60.6%; GP, 76.3%); 20.4% of neurologists would even check this at every visit. For this, 70.0% of neurologists and 63.4% of GPs used digital clinical support systems, which are usually integrated into the practice system.

NEU attributed more relevance to anticholinergic side effects for patient treatment (high relevance; NEU, 40.0%; GP, 21.3%; chi-squared-test, *p* < 0.001) and screened for these side effects significantly more often (NEU, 70.7%; GP, 44.4%; chi-squared-test, *p* < 0.001).

### 3.4 Interdisciplinary communication

The following findings can be found in Table 3. The interdisciplinary communication predominantly took place via the doctor’s letter (NEU, 85.7%; GP, 84.5%; chi-squared-test, *p* > 0.05). Other, considerably less common channels of interdisciplinary communication were phone calls (NEU, 35.4%; GP, 19.1%; chi-squared-test, *p* > 0.05) and the direct route via the patient (NEU, 39.5%; GP, 29.8%; chi-squared-test, *p* > 0.05).

**Table 3:** Aspects of interdisciplinary communication.

	Neurologists (*n* = 147)	General practitioners (*n* = 84)
Established arrangements for controls, *n* (%)		
Yes	29 (19.9)	16 (19.0)
No	117 (80.1)	68 (81.0)
Communication between practitioners, *n*		
Doctor’s letter	126	71
Telephone	52	16
Patient	58	25
Digital record	5	2
e-Mail	29	17
None	5	2
New medication notification, *n* (%)		
Yes	44 (31.9)	58 (71.6)***
No	94 (68.1)	23 (28.4)***
Communication of new medication, *n*		
Letter	24	60***
Telephone	2	2***
Patient	55	29***
Medication plan	43	36***

The questions marked in italics were multiple-choice questions. ***p < 0.001, chi-squared test. Significant differences are highlighted in bold.

Regarding the prescription of new medications, NEU were informed significantly less likely than GP (NEU, 31.9%; GP, 71.6%; chi-squared-test, *p* < 0.001). When NEU were informed about a new drug, it was usually through the patient or their medication plan (patient, 37.4%; medication plan, 29.3%). By contrast, GP generally received this information in a doctor’s letter (GP, 71.4%; NEU, 16.3%; chi-squared-test, *p* < 0.001).

The majority of NEU (80.1%) and GP (81.0%) stated that there were no formal arrangements for regular check-ups or medication issues between them.

## 4 Discussion

This questionnaire-based study was designed to investigate aspects of treatment safety of PD patients in Germany in an outpatient setting. It focused on monitoring mechanisms for drug safety and interdisciplinary communication between NEU and GP. Whilst, as expected, NEU treated a significantly higher number of PD patients, the number of geriatric Parkinson’s patients and the number of patients seen outside the outpatient clinic (home visits, nursing homes) was higher in GP. According to the majority of interviewed physicians of both specialties, there are no agreements regarding the conduction of monitoring procedures, essential for drug safety. ECG checks are thus commonly delegated by NEU, whereas laboratory tests are carried out independently of the GP. A large number of both professions showed an awareness of DDI and checked for them routinely at the very least when starting a new medication, usually with the use of an established digital clinical support system.

Providing safe and effective drug therapy for patients with PD is an immense challenge for the treating physicians (2, 17). This is due to several disease-and patient-specific factors: first, PD is a neurodegenerative multisystem disease that, in addition to the defining motor symptoms, has a number of non-motor symptoms, such as neuropsychiatric symptoms and autonomic dysfunction (18). Especially the latter, here for example dysphagia and delayed gastric emptying, should be emphasized in the context of pharmacokinetic aspects (19). The pharmacological treatment of motor symptoms is exceptionally complex and often requires a precise and timely administration of an explicit dose of the medication, sometimes spread across five or even more different times a day (20). This practical administration of drugs continues to become increasingly complex as the disease progresses and motor fluctuations arise, due to pathophysiological processes and the resulting altered response to dopaminergic stimulation (21). Hereby, a very tangible aspect of medication safety is brought to the fore, namely precise medication adherence. The probability of incorrect administration (forgotten doses, altered dosages) correlates with the frequency of intake times (21). In turn, ADR are more likely to occur, especially motor fluctuations or, if therapy is resumed spontaneously, an overdose of dopaminergic medication (22). The latter manifests itself in the typical symptoms of dopaminergic overstimulation, in particular psychoses, impulse control disorders and orthostatic hypotension (21). Thus, the treatment of motor symptoms alone poses considerable challenges for outpatient practitioners, not only in terms of therapeutic outcome but also with regard to maintaining adequate treatment safety. In addition, the non-motor symptoms significantly impact the patient’s quality of life and restrict them in activities of daily living, so that a drug therapy is usually necessary as well (23). Psychotic symptoms are a common non-motor symptom in the late stages of the PD. Clozapine is the only drug approved for this indication in Germany (24). However, clozapine is not only a drug that falls into the group of potentially inappropriate medications (PIMs) for the elderly, but it also carries a substantial risk of side effects and drug interactions (5, 25). The risk of clozapine-induced agranulocytosis is dramatically increased by combination with other drugs, such as the antidepressant mirtazapine or the antihypertensive ramiprile. The use of clozapine therefore requires strict long-term therapeutic drug monitoring, to name just one example among the pharmacological treatments of non-motor symptoms. Further, disease-specific factors, the patient demographics complicates the pharmacological therapy. PD usually affects elderly who in turn may already suffer from other comorbidities (2, 3, 5). Alongside the complex treatment of PD, a pharmacological treatment of mainly cardiovascular and neurological comorbid diseases is therefore required, which often results in polypharmacy and prescription of PIM for the elderly (5, 6, 26). Of particular hazard for PD patients are the aforementioned PIMs, which include, for example, benzodiazepines or tricyclic antidepressants such as amitriptyline (5). Besides the intrinsic unfavorable action in the elderly, e.g., the anticholinergic side effects with worsening of cognition of amitriptyline or dependence potential and drowsiness of benzodiazepines, these drugs often entail a high risk of DDI, especially with other central nervous system (CNS)-acting substances such as levodopa or amantadine (5). Finally, patients in this age group have significantly altered pharmacokinetics, which can influence the safety and, in particular, the effectiveness of the drugs (2, 8).

All of the listed factors, individually or in combination, increase the risk of ADR or DDI and thus pose a significant threat to patient safety (27). The aforementioned issues can, in the worst case, result in a hospital admission or prolonged stay and, rarely, even death (28–30). A meta-analysis even revealed that one in ten hospital admissions of patients over the age of 60 is attributable to an ADR or inappropriate medication (29). Such treatment errors are a major cost burden for the healthcare system (31).

In order to increase drug safety, especially in the vulnerable group of PD patients, and prevention of ADR and DDI, structured control mechanisms are necessary in addition to the awareness of the treating physician. These controls include screening for potential ADR through laboratory and apparative tests, as well as periodic checks for DDI and their subsequent regular monitoring (2). Laboratory assessments serve to identify contraindications (e.g., renal failure prior treatment with ropinirole) and side effects (e.g., liver failure caused by tolcapone), to assess the risk for potential side effects (e.g., electrolyte imbalance as a risk factor for cardiac arrhythmias), and to adjust dosages (e.g., dose adjustment of pramipexole with impaired kidney function). Consequently, laboratory tests with certain basic parameters (electrolytes, creatinine, liver enzymes, thyroid stimulating hormone, blood count and coagulation) should be performed before initiating a new drug therapy, regularly during the course of treatment (e.g., once a year) and in case of worsening clinical symptoms (2). In terms of apparatus-based tests, ECG is crucial. Recommended practice is to perform an ECG before starting specific PD-specific drugs (levodopa, apomorphine, amantadine, pimavanserin) in order to identify common comorbid arrhythmias as potential contraindications and to determine the QTc time (2, 5, 32). A prolongation of the latter is known to be a hazard factor for the occurrence of cardiac arrhythmias and should therefore be monitored regularly when using particular drugs, e.g., apomorphine and amantadine (2, 33, 34).

The majority of participating NEU (99.3%) and GP (91.4%) in our study stated that they carried out regular checks for DDIs, at least when a new medication is prescribed. That’s in line with suggestions in the literature, which recommends checking for DDIs when starting a new drug, when clinical symptoms get worse, and when there are signs that DDIs might be causing symptoms (e.g., psychosis) (2). Special consideration should be given to interactions with other CNS-active substances (antidepressants, opioids, benzodiazepines, neuroleptics), with other QTc-prolonging substances, and, especially in the case of COMT inhibitors, to interactions via cytochrome P450 enzymes (6). However, there is a reported lack of agreements regarding the resulting recommended monitoring tests to screen for specific DDI or other ADR (e.g., QTc prolongation, hyperkalemia), leading to gaps in medical care. One therapeutic gap affects, for example, the monitoring of anticholinergic side effects, particularly among GPs. Anticholinergic side effects, such as cognitive impairment, dizziness and bladder and bowel dysfunction, pose a particular threat to the highly vulnerable group of PD patients. The severity of motor and non-motor symptoms can be exacerbated by these anticholinergic side effects (35). In general, communication between treating physicians should be focused on key points. These include information about the initiation of a new drug or adjustment of a dosage, recommended follow-up examinations due to DDIs or to monitor ADR, and finally information regarding already conducted check-ups.

The question then arises as how to structure communication and allocate responsibilities in a practical manner. One approach is obvious: at the center of communication and treatment is always the patient. Thus, a seamless flow of information and allocation of responsibilities regarding the patient could be ensured by an individual electronic health record (EHR). The latest information provided here, in combination with the existing digital alert systems, offer a sufficient possibility to quickly and reliably recognize potential DDIs and ADRs. In addition, collected and outstanding diagnostic findings could be shared and tasks distributed, making time-consuming personal communication obsolete. The fact that such an EHR promotes interprofessional exchange, enhances transparency and closes information gaps was demonstrated by the use of such systems in hospitals in various countries for several years, also with regard to the treatment of PD patients (36, 37). Wu and colleagues provided a detailed overview of the options and setup of EHCs for PD patients utilized in the US (37). Goldin and colleagues additionally investigated the impact of using best practice alerts (BPAs) on the prescription of antidopaminergic drugs in PD patients in an American hospital. Antidopaminergic drugs can lead to a worsening of Parkinsonian symptoms and, in this context, to further complications and a longer hospital stay (38–40). The prescription of contraindicated medication was reduced by almost 30% using the BPA (39). By standardizing the available products and by educating the healthcare professionals, such systems offer a substantial opportunity to increase medication safety (37, 41). In Germany, comprehensive implementation of appropriate patient-centered, non-hospital-specific systems such as an individual EHR is still missing, and therefore specific studies on effectiveness of an individual EHR are missing too. In a project for the expansion of an EHR in the form of an electronic insurance card was undertaken in Berlin among asylum seekers. While the primary focus was not on drug safety aspects, the processes resulting from the introduction of the card ultimately led to a reduction in outpatient therapy costs (42). In the field of PD in particular, a web-based tool (MANAGE-PD, German version: Parkinson Check) was investigated that is intended to support the identification of advanced PD and thereby optimize care and referral of such patients to advanced treatment options (43).

Another possibility for improving interdisciplinary communication is the development of so-called multidisciplinary PD networks (44, 45). In these collaborations, all medical practitioners necessary for the holistic treatment of a PD patient are bundled, including not only physicians but also therapists (e.g., physiotherapist). These networks, of which there are now 15 in Germany, have been shown to increase connectivity between practitioners and thus the quality of treatment, which is reflected in an increased quality of life (46, 47). The implementation of drug safety aspects within these networks could achieve a seamless and coordinated dialogue and thereby enhance patient safety.

## 5 Limitations

Since the treatment of PD involves not only the mentioned disciplines NEU and GP, but also others, such as urologists, psychiatrists or gastroenterologists, and non-physician healthcare professionals, e.g., specially qualified PD nurses, the data do not fully reflect the complexity of interdisciplinary communication. This limitation highlights the collected results and the subsequent considerations all the more: the larger the network of treating physicians, the more frequently and intensely the described interface issues could arise regarding the drug safety. Unfortunately, the expected low response rate should also be noted, which limits the generalizability of the results. Further, the presumed consequences of inadequate interdisciplinary communication were not investigated in more detail, namely ADR and their potential consequences (e.g., hospitalization). In this study a selection bias leading to a higher response rate of physicians with specific interest in this topic cannot be excluded.

## 6 Conclusion

The presented data point to a considerably improvable communication structure between outpatient treating physicians of PD patients in Germany. In this context, not only specialized NEU played a role, but also GP, particularly in rural areas, in the field of geriatrics and outside outpatient clinic (home visits, nursing homes). Although the screening mechanisms for DDI were established, the resulting control procedures were often unknowingly delegated to each other, creating a potential risk of gaps regarding the drug safety. Standardized and comprehensive interdisciplinary information exchange strategies are urgently needed.

## Data Availability

The raw data supporting the conclusions of this article will be made available by the authors, without undue reservation.
